# 2-(3-Chloro-4-hydroxy­phen­yl)-*N*-(3,4-dimethoxy­pheneth­yl)acetamide

**DOI:** 10.1107/S1600536808013299

**Published:** 2008-05-10

**Authors:** Rohan A. Davis, Peter C. Healy

**Affiliations:** a Eskitis Institute for Cell and Molecular Therapies, Griffith University, Nathan, Brisbane 4111, Australia

## Abstract

The title compound, C_18_H_20_ClNO_4_, was synthesized during the generation of a combinatorial library based on the fungal natural product 3-chloro-4-hydroxy­phenyl­acetamide. It crystallizes as discrete mol­ecules linked by inter­molecular *C*(9) chains of N—H⋯O and O—H⋯O hydrogen bonds which in turn combine to form chains of *R*_2_^2^(20) rings.

## Related literature

For related literature, see: Bernstein *et al.* (1995 ▶); Davis *et al.* (2005 ▶, 2007 ▶).
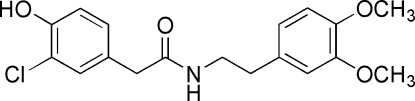


## Experimental

### 

#### Crystal data

C_18_H_20_ClNO_4_
*M* *_r_* = 349.80
Monoclinic, 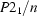

*a* = 12.329 (3) Å
*b* = 12.839 (5) Å
*c* = 11.062 (3) Å
β = 92.18 (2)°
*V* = 1749.8 (9) Å^3^
*Z* = 4
Mo *K*α radiation
μ = 0.24 mm^−1^
*T* = 295 K
0.35 × 0.35 × 0.15 mm

#### Data collection

Rigaku AFC-7R diffractometer
Absorption correction: none
3463 measured reflections
3078 independent reflections
1596 reflections with *I* > 2σ(*I*)
*R*_int_ = 0.034
3 standard reflections every 150 reflections intensity decay: 0.2%

#### Refinement

*R*[*F* ^2^ > 2σ(*F* ^2^)] = 0.056
*wR*(*F* ^2^) = 0.165
*S* = 0.99
3078 reflections
217 parameters
H-atom parameters constrained
Δρ_max_ = 0.21 e Å^−3^
Δρ_min_ = −0.31 e Å^−3^

### 

Data collection: *MSC/AFC7 Diffractometer Control for Windows* (Molecular Structure Corporation, 1999 ▶); cell refinement: *MSC/AFC7 Diffractometer Control for Windows*; data reduction: *TEXSAN for Windows* (Molecular Structure Corporation, 2001 ▶); program(s) used to solve structure: *TEXSAN for Windows*; program(s) used to refine structure: *TEXSAN for Windows* and *SHELXL97* (Sheldrick, 2008 ▶); molecular graphics: *ORTEP-3* (Farrugia, 1997 ▶); software used to prepare material for publication: *TEXSAN for Windows* and *PLATON* (Spek, 2003 ▶).

## Supplementary Material

Crystal structure: contains datablocks global, I. DOI: 10.1107/S1600536808013299/tk2268sup1.cif

Structure factors: contains datablocks I. DOI: 10.1107/S1600536808013299/tk2268Isup2.hkl

Additional supplementary materials:  crystallographic information; 3D view; checkCIF report

## Figures and Tables

**Table 1:** Hydrogen-bond geometry (Å, °)

*D*—H⋯*A*	*D*—H	H⋯*A*	*D*⋯*A*	*D*—H⋯*A*
N1—H1⋯O14^i^	0.91	2.37	3.066 (4)	134
O4—H4⋯O8^i^	0.90	1.74	2.616 (4)	165

Symmetry code: (i) 
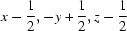
.

**Fig. 1.**
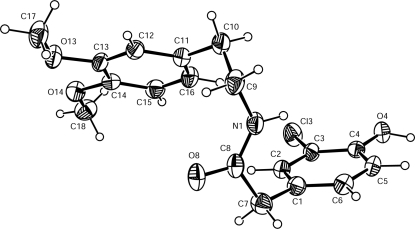
View of (I) with the atom numbering scheme. Displacement ellipsoids for non-H atoms are drawn at the 30% probability level.

**Fig. 2.**
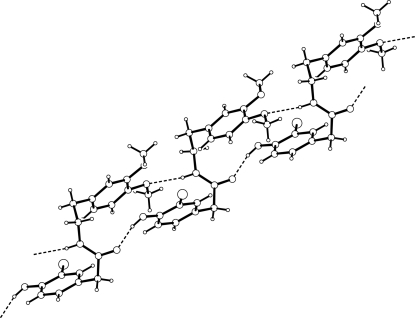
View of the intramolecular N—H···O and O—H···O hydrogen bonding (dashed lines) in (I).

## supplementary crystallographic information

### Comment

The title compound (I) was synthesized during the generation of a combinatorial library based on the fungal natural product 3-chloro-4-hydroxyphenylacetamide (Davis *et al.*, 2005, 2007). Compound (I) was shown to display moderate cytotoxicity towards the human melanoma cell line MM96L with 94% inhibition at 100 µg ml^-1^ (Davis *et al.*, 2007).

This compound crystallizes as discrete molecules with three planar components: the acetamide group (C7—C8(O8)—N1—C9), the 3-chloro-4-hydroxyphenyl group (C1—C7, O4, Cl3), and the 3,4-dimethoxybenzyl group (C11—C16, C10, O13—C17, O14—C18), Fig. 1. The C9—C10—C11—C16 and C2—C1—C7—C8 torsion angles are -91.8 (4)° and-78.9 (4)° respectively. In the crystal structure the amide (N1) and hydroxy (O4) groups form C(9) chains (Bernstein *et al.*, 1995) of intermolecular N—H···O and O—H···O hydrogen bonds with the methoxy oxygen (O14) and the carbonyl oxygen (O8), respectively (Table 1) which in turn combine to form chains of *R*_2_^2^(20) rings (Fig. 2).

### Experimental

Compound (I) was prepared as previously reported (Davis *et al.*, 2007). Crystals suitable for X-ray diffraction studies were obtained by slow evaporation of a n-hexane/ethyl acetate (1:1) solution of (I); m.p. 421–423 K.

### Refinement

The carbon-bound H atoms were constrained as riding atoms with C—H = 0.95–0.96 Å. The amide and hydroxyl protons were located in difference Fourier maps and constrained with N—H, O—H = 0.90 Å. *U*_iso_(H) values were set at 1.2*U*_eq_ of the parent atom.

### Crystal data

**Table:**

C_18_H_20_ClNO_4_	*F*_000_ = 736
*M**_r_* = 349.80	*D*_x_ = 1.328 Mg m^−^^3^
Monoclinic, *P*2_1_/*n*	Mo *K*α radiation λ = 0.7107 Å
Hall symbol: -P 2yn	Cell parameters from 25 reflections
*a* = 12.329 (3) Å	θ = 12.7–16.7º
*b* = 12.839 (5) Å	µ = 0.24 mm^−^^1^
*c* = 11.062 (3) Å	*T* = 295 K
β = 92.18 (2)º	Prismatic, colourless
*V* = 1749.8 (9) Å^3^	0.35 × 0.35 × 0.15 mm
*Z* = 4	

### Data collection

**Table:**

Rigaku AFC-7R diffractometer	*R*_int_ = 0.034
Radiation source: Rigaku rotating anode	θ_max_ = 25.0º
Monochromator: graphite	θ_min_ = 2.5º
*T* = 295 K	*h* = −14→6
ω–2θ scans	*k* = 0→15
Absorption correction: none	*l* = −13→13
3463 measured reflections	3 standard reflections
3078 independent reflections	every 150 reflections
1596 reflections with *I* > 2σ(*I*)	intensity decay: 0.2%

### Refinement

**Table:**

Refinement on *F*^2^	Secondary atom site location: difference Fourier map
Least-squares matrix: full	Hydrogen site location: inferred from neighbouring sites
*R*[*F*^2^ > 2σ(*F*^2^)] = 0.056	H-atom parameters constrained
*wR*(*F*^2^) = 0.165	*w* = 1/[σ^2^(*F*_o_^2^) + (0.0802*P*)^2^] where *P* = (*F*_o_^2^ + 2*F*_c_^2^)/3
*S* = 0.99	(Δ/σ)_max_ < 0.001
3078 reflections	Δρ_max_ = 0.21 e Å^−^^3^
217 parameters	Δρ_min_ = −0.31 e Å^−^^3^
Primary atom site location: structure-invariant direct methods	Extinction correction: none

### Special details

**Table:**

Experimental. The scan width was (1.68 + 0.30tanθ)° with an ω scan speed of 16° per minute (up to 4 scans to achieve I/σ(I) > 10). Stationary background counts were recorded at each end of the scan, and the scan time:background time ratio was 2:1.
Geometry. Bond distances, angles *etc*. have been calculated using the rounded fractional coordinates. All su's are estimated from the variances of the (full) variance-covariance matrix. The cell e.s.d.'s are taken into account in the estimation of distances, angles and torsion angles
Refinement. Refinement of *F*^2^ against ALL reflections. The weighted *R*-factor *wR* and goodness of fit *S* are based on *F*^2^, conventional *R*-factors *R* are based on *F*, with *F* set to zero for negative *F*^2^. The threshold expression of *F*^2^ > 2σ(*F*^2^) is used only for calculating *R*-factors(gt) *etc*. and is not relevant to the choice of reflections for refinement. *R*-factors based on *F*^2^ are statistically about twice as large as those based on *F*, and *R*- factors based on ALL data will be even larger.

### Fractional atomic coordinates and isotropic or equivalent isotropic displacement parameters (Å^2^)

**Table:**

	*x*	*y*	*z*	*U*_iso_*/*U*_eq_	
Cl3	0.45169 (9)	0.55050 (8)	0.30062 (9)	0.0775 (4)	
O4	0.28934 (19)	0.46680 (18)	0.13154 (19)	0.0576 (8)	
O8	0.5970 (2)	0.0875 (2)	0.5455 (2)	0.0730 (10)	
O13	1.02796 (18)	0.23926 (19)	0.5658 (2)	0.0617 (9)	
O14	0.94460 (19)	0.41834 (18)	0.6082 (2)	0.0596 (9)	
N1	0.5696 (2)	0.1267 (2)	0.3474 (3)	0.0582 (11)	
C1	0.3920 (3)	0.2566 (3)	0.3989 (3)	0.0520 (12)	
C2	0.4348 (3)	0.3564 (3)	0.3927 (3)	0.0531 (14)	
C3	0.3990 (3)	0.4245 (3)	0.3042 (3)	0.0488 (12)	
C4	0.3205 (3)	0.3961 (3)	0.2174 (3)	0.0449 (11)	
C5	0.2765 (3)	0.2977 (3)	0.2247 (3)	0.0506 (12)	
C6	0.3118 (3)	0.2284 (3)	0.3136 (3)	0.0544 (12)	
C7	0.4368 (3)	0.1806 (3)	0.4917 (3)	0.0640 (16)	
C8	0.5425 (3)	0.1284 (3)	0.4612 (4)	0.0555 (12)	
C9	0.6665 (3)	0.0772 (3)	0.3040 (4)	0.0674 (16)	
C10	0.7448 (3)	0.1555 (3)	0.2523 (3)	0.0669 (14)	
C11	0.7947 (3)	0.2290 (3)	0.3448 (3)	0.0541 (14)	
C12	0.8890 (3)	0.1991 (3)	0.4103 (3)	0.0527 (12)	
C13	0.9362 (3)	0.2626 (3)	0.4968 (3)	0.0488 (11)	
C14	0.8904 (3)	0.3599 (3)	0.5209 (3)	0.0506 (11)	
C15	0.7965 (3)	0.3898 (3)	0.4579 (3)	0.0576 (12)	
C16	0.7501 (3)	0.3244 (3)	0.3708 (3)	0.0613 (14)	
C17	1.0790 (3)	0.1422 (3)	0.5422 (4)	0.0773 (17)	
C18	0.8999 (3)	0.5175 (3)	0.6355 (4)	0.0728 (17)	
H1	0.52920	0.15340	0.28470	0.0690*	
H2	0.48940	0.37790	0.45040	0.0640*	
H4	0.22920	0.44390	0.09130	0.0690*	
H5	0.22100	0.27710	0.16780	0.0600*	
H6	0.28090	0.16070	0.31640	0.0650*	
H7A	0.44830	0.21720	0.56580	0.0770*	
H7B	0.38400	0.12770	0.50170	0.0770*	
H9A	0.70220	0.04160	0.36940	0.0810*	
H9B	0.64580	0.02860	0.24260	0.0810*	
H10A	0.80160	0.11820	0.21600	0.0800*	
H10B	0.70660	0.19560	0.19240	0.0800*	
H12	0.92100	0.13350	0.39450	0.0630*	
H15	0.76400	0.45500	0.47430	0.0690*	
H16	0.68580	0.34580	0.32790	0.0730*	
H17A	1.14550	0.13770	0.58820	0.0930*	
H17B	1.03260	0.08680	0.56430	0.0930*	
H17C	1.09290	0.13730	0.45860	0.0930*	
H18A	0.82940	0.50840	0.66600	0.0870*	
H18B	0.94520	0.55140	0.69460	0.0870*	
H18C	0.89500	0.55850	0.56410	0.0870*	

### Atomic displacement parameters (Å^2^)

**Table:**

	*U*^11^	*U*^22^	*U*^33^	*U*^12^	*U*^13^	*U*^23^
Cl3	0.0899 (8)	0.0688 (7)	0.0723 (7)	−0.0335 (6)	−0.0161 (5)	0.0010 (5)
O4	0.0595 (15)	0.0560 (15)	0.0560 (14)	−0.0045 (12)	−0.0141 (12)	0.0018 (12)
O8	0.0645 (17)	0.0801 (19)	0.0720 (17)	0.0059 (15)	−0.0273 (14)	0.0085 (15)
O13	0.0508 (15)	0.0643 (17)	0.0686 (16)	0.0122 (13)	−0.0165 (12)	−0.0127 (13)
O14	0.0601 (16)	0.0540 (16)	0.0643 (15)	0.0047 (13)	−0.0015 (12)	−0.0106 (12)
N1	0.0493 (19)	0.067 (2)	0.057 (2)	0.0096 (16)	−0.0136 (15)	−0.0031 (16)
C1	0.050 (2)	0.052 (2)	0.054 (2)	0.0073 (18)	0.0011 (17)	0.0026 (17)
C2	0.045 (2)	0.064 (3)	0.050 (2)	−0.0033 (18)	−0.0018 (16)	−0.0048 (18)
C3	0.047 (2)	0.049 (2)	0.050 (2)	−0.0101 (17)	−0.0029 (16)	−0.0042 (17)
C4	0.0438 (19)	0.045 (2)	0.0461 (19)	0.0037 (17)	0.0030 (15)	−0.0058 (16)
C5	0.040 (2)	0.050 (2)	0.061 (2)	−0.0008 (16)	−0.0082 (17)	−0.0053 (17)
C6	0.053 (2)	0.045 (2)	0.065 (2)	0.0008 (17)	0.0003 (18)	−0.0048 (18)
C7	0.069 (3)	0.063 (3)	0.060 (2)	0.012 (2)	0.0017 (19)	0.0085 (19)
C8	0.056 (2)	0.047 (2)	0.062 (2)	−0.0032 (18)	−0.017 (2)	−0.0027 (19)
C9	0.056 (2)	0.066 (3)	0.079 (3)	0.014 (2)	−0.013 (2)	−0.018 (2)
C10	0.051 (2)	0.091 (3)	0.058 (2)	0.013 (2)	−0.0077 (18)	−0.010 (2)
C11	0.045 (2)	0.068 (3)	0.049 (2)	0.0019 (19)	−0.0020 (16)	−0.0011 (18)
C12	0.045 (2)	0.060 (2)	0.053 (2)	0.0025 (17)	0.0000 (17)	−0.0080 (18)
C13	0.0361 (19)	0.056 (2)	0.054 (2)	0.0038 (17)	−0.0033 (16)	0.0037 (17)
C14	0.047 (2)	0.058 (2)	0.0474 (19)	−0.0014 (18)	0.0082 (16)	0.0020 (18)
C15	0.055 (2)	0.057 (2)	0.061 (2)	0.0103 (19)	0.0056 (19)	0.0072 (19)
C16	0.048 (2)	0.072 (3)	0.063 (2)	0.010 (2)	−0.0076 (18)	0.008 (2)
C17	0.065 (3)	0.078 (3)	0.087 (3)	0.025 (2)	−0.022 (2)	−0.017 (2)
C18	0.071 (3)	0.065 (3)	0.083 (3)	0.004 (2)	0.010 (2)	−0.013 (2)

### Geometric parameters (Å, °)

**Table:**

Cl3—C3	1.744 (4)	C12—C13	1.370 (5)
O4—C4	1.359 (4)	C13—C14	1.401 (5)
O8—C8	1.245 (5)	C14—C15	1.383 (5)
O13—C13	1.374 (4)	C15—C16	1.385 (5)
O13—C17	1.425 (5)	C2—H2	0.9500
O14—C14	1.376 (4)	C5—H5	0.9500
O14—C18	1.424 (5)	C6—H6	0.9500
O4—H4	0.9000	C7—H7A	0.9500
N1—C9	1.451 (5)	C7—H7B	0.9500
N1—C8	1.315 (5)	C9—H9A	0.9500
N1—H1	0.9100	C9—H9B	0.9500
C1—C2	1.388 (5)	C10—H10A	0.9500
C1—C7	1.506 (5)	C10—H10B	0.9500
C1—C6	1.389 (5)	C12—H12	0.9500
C2—C3	1.373 (5)	C15—H15	0.9500
C3—C4	1.386 (5)	C16—H16	0.9500
C4—C5	1.379 (5)	C17—H17A	0.9500
C5—C6	1.384 (5)	C17—H17B	0.9500
C7—C8	1.515 (5)	C17—H17C	0.9500
C9—C10	1.520 (5)	C18—H18A	0.9500
C10—C11	1.506 (5)	C18—H18B	0.9500
C11—C16	1.377 (5)	C18—H18C	0.9500
C11—C12	1.400 (5)		
			
Cl3···O4	2.894 (3)	C14···H18C^vii^	3.0300
Cl3···C12^i^	3.646 (4)	C15···H18A	2.7800
Cl3···H12^i^	2.9200	C15···H9B^i^	2.9500
Cl3···H17C^i^	3.1100	C15···H18C	2.7300
Cl3···H2^ii^	2.9700	C17···H12	2.5000
O4···Cl3	2.894 (3)	C17···H10B^iv^	3.0600
O4···C17^i^	3.411 (5)	C18···H15	2.5300
O4···O8^iii^	2.616 (4)	H1···C1	2.5200
O8···C4^iv^	3.295 (4)	H1···C6	2.8800
O8···C5^iv^	3.266 (5)	H1···H10B	2.5100
O8···O4^iv^	2.616 (4)	H1···O13^iii^	2.7900
O8···C8^v^	3.262 (5)	H1···O14^iii^	2.3700
O13···O14	2.569 (3)	H2···H7A	2.4900
O14···C6^iv^	3.417 (4)	H2···Cl3^ii^	2.9700
O14···O13	2.569 (3)	H4···H5	2.3100
O14···N1^iv^	3.066 (4)	H4···O8^iii^	1.7400
O4···H17C^i^	2.8300	H4···C8^iii^	2.8200
O4···H18A^ii^	2.7400	H4···H9A^iii^	2.4700
O4···H6^vi^	2.7100	H5···H4	2.3100
O8···H9A	2.4500	H5···O8^iii^	2.6500
O8···H4^iv^	1.7400	H6···H7B	2.4100
O8···H7B^v^	2.8200	H6···O4^ix^	2.7100
O8···H5^iv^	2.6500	H7A···H2	2.4900
O13···H1^iv^	2.7900	H7B···H6	2.4100
O13···H10B^iv^	2.7000	H7B···O8^v^	2.8200
O14···H18C^vii^	2.8100	H9A···O8	2.4500
O14···H1^iv^	2.3700	H9A···C12	3.0900
N1···C2	3.431 (5)	H9A···H4^iv^	2.4700
N1···C6	3.443 (5)	H9B···C15^viii^	2.9500
N1···C16	3.379 (5)	H10A···H12	2.4300
N1···O14^iii^	3.066 (4)	H10B···H1	2.5100
C2···N1	3.431 (5)	H10B···H16	2.4600
C4···C18^ii^	3.406 (5)	H10B···O13^iii^	2.7000
C4···O8^iii^	3.295 (4)	H10B···C17^iii^	3.0600
C4···C13^iii^	3.522 (5)	H10B···H17A^iii^	2.5300
C5···C14^iii^	3.375 (5)	H12···C17	2.5000
C5···C13^iii^	3.348 (5)	H12···H10A	2.4300
C5···O8^iii^	3.266 (5)	H12···H17B	2.3600
C6···C14^iii^	3.598 (5)	H12···H17C	2.2100
C6···N1	3.443 (5)	H12···Cl3^viii^	2.9200
C6···O14^iii^	3.417 (4)	H15···C18	2.5300
C8···C8^v^	3.574 (6)	H15···H18A	2.3400
C8···O8^v^	3.262 (5)	H15···H18C	2.2900
C12···Cl3^viii^	3.646 (4)	H16···H10B	2.4600
C13···C4^iv^	3.522 (5)	H17A···H10B^iv^	2.5300
C13···C5^iv^	3.348 (5)	H17B···C12	2.8100
C14···C18^vii^	3.535 (6)	H17B···H12	2.3600
C14···C6^iv^	3.598 (5)	H17C···C12	2.6700
C14···C5^iv^	3.375 (5)	H17C···H12	2.2100
C16···N1	3.379 (5)	H17C···Cl3^viii^	3.1100
C17···O4^viii^	3.411 (5)	H17C···O4^viii^	2.8300
C18···C4^ii^	3.406 (5)	H18A···C15	2.7800
C18···C14^vii^	3.535 (6)	H18A···H15	2.3400
C1···H1	2.5200	H18A···O4^ii^	2.7400
C3···H18A^ii^	2.9800	H18A···C3^ii^	2.9800
C4···H18A^ii^	2.6000	H18A···C4^ii^	2.6000
C5···H18A^ii^	3.0800	H18A···C5^ii^	3.0800
C6···H1	2.8800	H18C···C15	2.7300
C8···H4^iv^	2.8200	H18C···H15	2.2900
C12···H17B	2.8100	H18C···O14^vii^	2.8100
C12···H17C	2.6700	H18C···C14^vii^	3.0300
C12···H9A	3.0900		
			
C13—O13—C17	116.7 (3)	C4—C5—H5	119.00
C14—O14—C18	117.1 (3)	C6—C5—H5	119.00
C4—O4—H4	109.00	C1—C6—H6	120.00
C8—N1—C9	124.5 (3)	C5—C6—H6	120.00
C9—N1—H1	110.00	C1—C7—H7A	108.00
C8—N1—H1	125.00	C1—C7—H7B	108.00
C2—C1—C7	120.1 (3)	C8—C7—H7A	108.00
C6—C1—C7	121.8 (3)	C8—C7—H7B	108.00
C2—C1—C6	118.0 (3)	H7A—C7—H7B	109.00
C1—C2—C3	120.7 (3)	N1—C9—H9A	109.00
Cl3—C3—C2	119.8 (3)	N1—C9—H9B	109.00
Cl3—C3—C4	118.6 (3)	C10—C9—H9A	109.00
C2—C3—C4	121.6 (4)	C10—C9—H9B	109.00
O4—C4—C3	118.7 (3)	H9A—C9—H9B	109.00
O4—C4—C5	123.5 (3)	C9—C10—H10A	108.00
C3—C4—C5	117.8 (3)	C9—C10—H10B	108.00
C4—C5—C6	121.1 (3)	C11—C10—H10A	108.00
C1—C6—C5	120.8 (4)	C11—C10—H10B	108.00
C1—C7—C8	115.5 (3)	H10A—C10—H10B	109.00
O8—C8—N1	124.1 (3)	C11—C12—H12	119.00
O8—C8—C7	117.7 (4)	C13—C12—H12	119.00
N1—C8—C7	118.2 (3)	C14—C15—H15	120.00
N1—C9—C10	112.2 (3)	C16—C15—H15	120.00
C9—C10—C11	114.1 (3)	C11—C16—H16	119.00
C10—C11—C16	122.9 (3)	C15—C16—H16	119.00
C10—C11—C12	119.3 (3)	O13—C17—H17A	109.00
C12—C11—C16	117.8 (3)	O13—C17—H17B	109.00
C11—C12—C13	121.4 (4)	O13—C17—H17C	110.00
O13—C13—C12	125.3 (3)	H17A—C17—H17B	109.00
O13—C13—C14	114.7 (3)	H17A—C17—H17C	109.00
C12—C13—C14	120.0 (3)	H17B—C17—H17C	109.00
O14—C14—C15	125.3 (3)	O14—C18—H18A	109.00
O14—C14—C13	115.6 (3)	O14—C18—H18B	109.00
C13—C14—C15	119.2 (3)	O14—C18—H18C	109.00
C14—C15—C16	119.9 (4)	H18A—C18—H18B	109.00
C11—C16—C15	121.8 (3)	H18A—C18—H18C	109.00
C1—C2—H2	120.00	H18B—C18—H18C	110.00
C3—C2—H2	120.00		
			
C17—O13—C13—C12	1.8 (5)	C3—C4—C5—C6	−1.9 (5)
C17—O13—C13—C14	−178.4 (3)	C4—C5—C6—C1	0.7 (6)
C18—O14—C14—C13	−179.7 (3)	C1—C7—C8—O8	161.4 (3)
C18—O14—C14—C15	0.1 (5)	C1—C7—C8—N1	−20.3 (5)
C9—N1—C8—O8	0.0 (6)	N1—C9—C10—C11	66.8 (4)
C9—N1—C8—C7	−178.2 (3)	C9—C10—C11—C12	86.5 (4)
C8—N1—C9—C10	−115.1 (4)	C9—C10—C11—C16	−91.8 (4)
C6—C1—C2—C3	−0.4 (5)	C10—C11—C12—C13	−178.9 (3)
C7—C1—C2—C3	176.3 (3)	C16—C11—C12—C13	−0.5 (5)
C2—C1—C6—C5	0.5 (5)	C10—C11—C16—C15	178.8 (3)
C7—C1—C6—C5	−176.1 (3)	C12—C11—C16—C15	0.5 (5)
C2—C1—C7—C8	−78.9 (4)	C11—C12—C13—O13	179.5 (3)
C6—C1—C7—C8	97.7 (4)	C11—C12—C13—C14	−0.3 (5)
C1—C2—C3—Cl3	178.4 (3)	O13—C13—C14—O14	1.0 (4)
C1—C2—C3—C4	−0.9 (6)	O13—C13—C14—C15	−178.7 (3)
Cl3—C3—C4—O4	1.3 (5)	C12—C13—C14—O14	−179.2 (3)
Cl3—C3—C4—C5	−177.3 (3)	C12—C13—C14—C15	1.1 (5)
C2—C3—C4—O4	−179.4 (3)	O14—C14—C15—C16	179.2 (3)
C2—C3—C4—C5	2.0 (5)	C13—C14—C15—C16	−1.1 (5)
O4—C4—C5—C6	179.5 (3)	C14—C15—C16—C11	0.3 (5)

Symmetry codes: (i) −*x*+3/2, *y*+1/2, −*z*+1/2; (ii) −*x*+1, −*y*+1, −*z*+1; (iii) *x*−1/2, −*y*+1/2, *z*−1/2; (iv) *x*+1/2, −*y*+1/2, *z*+1/2; (v) −*x*+1, −*y*, −*z*+1; (vi) −*x*+1/2, *y*+1/2, −*z*+1/2; (vii) −*x*+2, −*y*+1, −*z*+1; (viii) −*x*+3/2, *y*−1/2, −*z*+1/2; (ix) −*x*+1/2, *y*−1/2, −*z*+1/2.

### Hydrogen-bond geometry (Å, °)

**Table:**

*D*—H···*A*	*D*—H	H···*A*	*D*···*A*	*D*—H···*A*
N1—H1···O14^iii^	0.91	2.37	3.066 (4)	134
O4—H4···O8^iii^	0.90	1.74	2.616 (4)	165

Symmetry codes: (iii) *x*−1/2, −*y*+1/2, *z*−1/2.
